# The Relationship between Abdominal Fat Phenotypes and Insulin Resistance in Non-Obese Individuals after Acute Pancreatitis

**DOI:** 10.3390/nu12092883

**Published:** 2020-09-21

**Authors:** Juyeon Ko, Loren Skudder-Hill, Jaelim Cho, Sakina H. Bharmal, Maxim S. Petrov

**Affiliations:** School of Medicine, University of Auckland, Auckland 1023, New Zealand; ju.ko@auckland.ac.nz (J.K.); loren7891@outlook.com (L.S.-H.); j.cho@auckland.ac.nz (J.C.); s.bharmal@auckland.ac.nz (S.H.B.)

**Keywords:** diabetes, pancreatitis, insulin resistance, intra-pancreatic fat, visceral fat, fat phenotypes, lipid metabolism

## Abstract

Both type 2 prediabetes/diabetes (T2DM) and new-onset prediabetes/diabetes after acute pancreatitis (NODAP) are characterized by impaired tissue sensitivity to insulin action. Although the outcomes of NODAP and T2DM are different, it is unknown whether drivers of insulin resistance are different in the two types of diabetes. This study aimed to investigate the associations between abdominal fat phenotypes and indices of insulin sensitivity in non-obese individuals with NODAP, T2DM, and healthy controls. Indices of insulin sensitivity (homeostasis model assessment of insulin sensitivity (HOMA-IS), Raynaud index, triglyceride and glucose (TyG) index, Matsuda index) were calculated in fasting and postprandial states. Fat phenotypes (intra-pancreatic fat, intra-hepatic fat, skeletal muscle fat, visceral fat, and subcutaneous fat) were determined using magnetic resonance imaging and spectroscopy. Linear regression and relative importance analyses were conducted. Age, sex, and glycated hemoglobin A1c were adjusted for. A total of 78 non-obese individuals (26 NODAP, 20 T2DM, and 32 healthy controls) were included. Intra-pancreatic fat was significantly associated with all the indices of insulin sensitivity in the NODAP group, consistently in both the unadjusted and adjusted models. Intra-pancreatic fat was not significantly associated with any index of insulin sensitivity in the T2DM and healthy controls groups. The variance in HOMA-IS was explained the most by intra-pancreatic fat (R^2^ = 29%) in the NODAP group and by visceral fat (R^2^ = 21%) in the T2DM group. The variance in the Raynaud index was explained the most by intra-pancreatic fat (R^2^ = 18%) in the NODAP group and by visceral fat (R^2^ = 15%) in the T2DM group. The variance in the TyG index was explained the most by visceral fat in both the NODAP group (R^2^ = 49%) and in the T2DM group (R^2^ = 25%). The variance in the Matsuda index was explained the most by intra-pancreatic fat (R^2^ = 48%) in the NODAP group and by visceral fat (R^2^ = 38%) in the T2DM group. The differing association between intra-pancreatic fat and insulin resistance can be used to differentiate NODAP from T2DM. Insulin resistance in NODAP appears to be predominantly driven by increased intra-pancreatic fat deposition.

## 1. Introduction

Acute pancreatitis (AP) is an inflammatory process in the pancreas that may remain localized, spread to nearby tissues, or lead to systemic inflammation. Initially thought to be mainly a self-resolving disease, emerging evidence has shown that individuals after AP often develop metabolic derangements after hospital discharge—in particular, new-onset prediabetes or diabetes after AP (NODAP) [1,2,3,4,5,6]. For example, a 2020 prospective longitudinal cohort study (as part of the LACERTA project) demonstrated that 43% of non-diabetic patients with AP developed NODAP within two years after hospital discharge [7]. There is a growing appreciation that NODAP is different from type 2 prediabetes or diabetes mellitus (T2DM). A 2020 case-control study (as part of the MENSA project) showed that both fasting and postprandial levels of oxyntomodulin—a gut hormone involved in the regulation of exocrine pancreatic function—were significantly lower in NODAP than T2DM [8]. There are also several lines of epidemiological evidence that showed that outcomes of NODAP and T2DM are different. These include the risks of poor glycemic control [9], pancreatic cancer [10], mortality [11], and the risk–benefit ratio of common antidiabetic medications [12,13]. Hence, a thorough understanding of the mechanisms behind impaired glucose metabolism in patients after AP is critical with a view to improving clinical management and identifying potential novel targets for prevention and treatment.

Excess adiposity is strongly associated with the development of insulin resistance, which plays a key role in the pathogenesis of both T2DM and NODAP. The notion that general adiposity (as measured by body mass index (BMI)) worsens the course of AP was introduced nearly three decades ago [14] and has been confirmed in a 2020 meta-analysis [15]. Further, abdominal adiposity has been recognized as an even more important player than general adiposity in the setting of AP [16,17]. Implications of ectopic fat deposition in skeletal muscle, liver, and pancreas on insulin resistance have also become appreciated [18,19,20]. However, the effect of abdominal adiposity and ectopic fat phenotypes on insulin resistance in recent studies might have been confounded by a high BMI. Emerging evidence indicates that obese (i.e., BMI more than 30 kg/m^2^) and non-obese people with diabetes have different metabolic profiles. For example, a 2020 clinical study investigating insulin sensitivity (with the use of a hyperinsulinemic–euglycemic clamp) of non-obese versus obese individuals with T2DM demonstrated that insulin sensitivity (determined by the ratio of metabolic rate to steady-state insulin) was more than two-times greater in non-obese than obese individuals with T2DM (*p* < 0.05) [21]. Another clinical study using a clamp technique to investigate glucose metabolism in non-obese versus obese individuals with T2DM also showed significant differences in insulin sensitivity (i.e., insulin sensitivity index, *p* < 0.01) and insulin secretion (i.e., fasting C-peptide response, *p* < 0.05) [22]. To the best of our knowledge, to date, no study has investigated the implications of abdominal adiposity and ectopic fat phenotypes on insulin sensitivity specifically in non-obese individuals with NODAP.

The present study aimed to investigate the associations between magnetic resonance (MR) imaging-derived abdominal fat phenotypes (i.e., intra-pancreatic fat deposition (IPFD), intra-hepatic fat deposition (IHFD), skeletal muscle fat deposition (SMFD), visceral fat volume (VFV), subcutaneous fat volume (SFV)) and indices of insulin sensitivity in non-obese individuals with NODAP, T2DM, and healthy controls.

## 2. Materials and Methods

### 2.1. Study Population

This cross-sectional study nested into a prospective cohort study was conducted as part of the ARIES project and was approved by the Health and Disability Ethics Committee [23,24,25,26]. From the prospective cohort, non-obese (BMI < 30.0 kg/m^2^) adults with T2DM prior to AP and non-obese individuals with NODAP were identified. Individuals with fasting plasma glucose ≥100 mg/dL (≥5.6 mmol/L) and/or glycated hemoglobin A1c (HbA1c) ≥5.7% (39 mmol/mol) beyond three months of hospital discharge for AP constituted the NODAP group, in line with the published recommendations [27,28]. Individuals with HbA1c ≥5.7% (39 mmol/mol) before, during hospitalization for AP, or within three months after constituted the T2DM group. Fasting plasma glucose ≥100 mg/dL (≥5.6 mmol/L) during hospitalization was not considered as an eligibility criterion for T2DM due to the possibility of stress-induced hyperglycemia during acute illness [29]. All cases were at least 18 years old, provided informed consent, had a primary diagnosis of mild AP established prospectively at the time of hospitalization according to the international guidelines [30], and met the American Diabetes Association criteria for prediabetes or diabetes [31].

Individuals were excluded from the study if they had a recurrent attack of AP within three months prior to enrolment in the study, chronic pancreatitis, post-endoscopic retrograde cholangiopancreatography pancreatitis, fluid collections, pancreatic lipomatosis or lipomatous pseudohypertrophy, congenital anomalies of the pancreas or hereditary pancreatitis, cystic fibrosis, malignancy, cognitive disability, received surgical, endoscopic or radiological interventions involving the pancreas, had metallic foreign body implantations heart pacemakers, or other electronic device implantations, received steroid therapy, or were pregnant.

Healthy controls were also recruited. They were at least 18 years old, provided informed consent, had a BMI less than 30.0 kg/m^2^, no personal and family history of diseases of the exocrine pancreas and diabetes, no family history of cystic fibrosis or coeliac diseases, no cancer, no upper abdominal symptoms in the 12 months preceding the study, and no history or evaluation for infectious or inflammatory diseases in the 6 months preceding the study.

### 2.2. Clinic Visit

The study participants were invited to attend the COSMOS clinic at the University of Auckland (New Zealand) to undergo a mixed meal test. After an overnight fast of at least 8 h, a venous catheter with stopcock apparatus was inserted into each participant’s arm for serial blood sample collection. Participants consumed a commercially available mixed meal drink (BOOST Original, Nestlé Health Science, Bridgewater, NJ, USA) providing 61.5 g carbohydrates, 15 g protein, and 6 g fat. All blood samples were centrifuged at 4000× *g* for 5 min at 4 °C, serum was separated into aliquots and stored at −80 °C until use. Anthropometric data (height and weight) of all eligible participants were recorded to calculate BMI. All measurements were taken over the light clothing of participants, and height and weight were measured in a standing position without shoes and headgear. 

### 2.3. Quantification of Abdominal Fat Phenotypes

#### 2.3.1. Imaging Protocol

Abdominal MR imaging for all participants was performed at the University of Auckland, using a 3.0 Tesla MAGNETOM Skyra scanner (Siemens, Erlangen, Germany). Participants were asked to lie down and hold their breath at end-expiration. An Axial T1-weighted volumetric interpolated breath-hold examination Dixon sequence was applied with the following parameters: true form abdomen shim mode; field-of-view, 420 mm; base resolution, 320; echo time, 1.27 ms, 2.5 ms; repetition time, 3.85 ms; flip angle, 9°; pixel bandwidth, 920 Hz; slice thickness, 5 mm. For each participant, IPFD, IHFD, SMFD, VFV, and SFV were quantified using ImageJ software (National Institutes of Health, Bethesda, MD, USA). Fat phenotypes were quantified independently by two raters, and average values of two independent measurements were used for statistical analyses.

#### 2.3.2. Intra-Pancreatic Fat Deposition

IPFD (%) was measured using the “MR-opsy” method, a chemical shift MR imaging technique that provides unambiguous water–fat signal separation on in-phase and out-of-phase images [26,32,33]. Two slices with clear visualization of the entire pancreas were selected from a series of MR scans. Three regions of interest were placed in the head, body, and tail of the pancreas for estimation of IPFD (%). To prevent the inclusion of non-parenchymal tissues within each region of interest, 1–20% thresholding was applied in line with the published recommendations [33]. IPFD (%) was calculated as the average pancreatic fat fraction of both slices.

#### 2.3.3. Intra-Hepatic Fat Deposition

Single-voxel MR spectroscopy was used to determine IHFD (%). A voxel (20 × 20 × 20 mm) was positioned in the right lobe of the liver, away from the blood vessels and bile ducts and at least 10 mm away from the edge. Automated shimming was performed prior to signal acquisition to improve B0 homogeneity. Spectra were acquired using a free-breathing navigator-triggered spin echo acquisition with repetition time ≥3000 ms, echo time = 33 ms, 50 averages. Acquisition duration was 853 ms. Both water-suppressed and non-water-suppressed spectra were taken, with the non-water-suppressed spectrum acting as the reference for IHFD quantification. Spectra were processed and analyzed using SIVIC software (University of California, San Francisco, CA, USA) [34]. Fat fraction was defined as the area under fat (methylene (CH2)) peak divided by area under fat and water peaks multiplied by 100%.

#### 2.3.4. Skeletal Muscle Fat Deposition

Total muscle area and intra-muscular fat area of erector spinae muscles were measured using a single axial slice at the lower endplate of L3 vertebra, in line with published recommendations [35]. The free-hand tool of ImageJ software was used to outline the left and right erector spinae muscles followed by a measurement of the total pixel content [35,36,37]. Further, to calculate intra-muscular fat area, the threshold function of ImageJ was used to convert grayscale pixels into binary images, using the global histogram-derived method. Care was taken to exclude extra-muscular fat (i.e., beyond the fascial layer of the erector spinae muscles). Total muscle area and intra-muscular fat area were calculated by multiplying the selected total pixel content with the pixel surface area. Ratio of fat-free cross-sectional muscle area to total cross-sectional muscle area was determined by subtracting intra-muscular fat area from total muscle area and dividing the resulting value by total muscle area. SMFD (%) was calculated as (1 − fat-free cross-sectional muscle area to total cross-sectional muscle area ratio) × 100%.

#### 2.3.5. Subcutaneous and Visceral Fat Volumes

Quantification of SFV and VFV was conducted from the second to the fifth lumbar levels by segmentation of subcutaneous and visceral fat compartments using the free-hand tool of ImageJ [38,39]. Using the free-hand tool, visceral and subcutaneous fat regions were delineated and measured separately. Non-adipose tissues, soft organs, and blood vessels were excluded from the measurement of visceral fat. The final step for all the above measurements involved the summation of the pixel contents of all the slices in series, multiplying by the slice thickness and pixel area to obtain the total volume.

### 2.4. Laboratory Data

HbA1c, plasma glucose, and insulin were analyzed at LabPlus—tertiary referral medical laboratory at Auckland City Hospital (Auckland, New Zealand). HbA1c was measured using the boronate affinity chromatography assay (Trinity Biotech, Wicklow, Ireland). Plasma glucose was measured using the enzymatic colorimetric assay (F.Hoffmann-La Roche Ltd., Basel, Switzerland). Insulin was measured using a chemiluminescence sandwich immunoassay (Roche Diagnostics, Auckland, New Zealand). The same laboratory measured lipid profile (triglycerides, total cholesterol, HDL cholesterol, and LDL cholesterol).

### 2.5. Indices of Insulin Sensitivity 

The following 4 indices of insulin sensitivity were studied: homeostasis model assessment of insulin sensitivity (HOMA-IS), the Raynaud index, and the triglyceride and glucose (TyG) index in fasting state; as well as the Matsuda index in postprandial state [40,41]. HOMA-IS was calculated as 1/HOMA-IR, with HOMA-IR values obtained using the HOMA2 calculator (version 2.2.3 Diabetes Trials Unit, University of Oxford, Oxford, UK). The Raynaud index was calculated using the formula 40/Ins_0_, where Ins_0_ represented the insulin value in fasting state. The Matsuda index was calculated from a mixed meal test (as described elsewhere [40]) using the formula: 10,000/$\sqrt{\left({\mathrm{Glu}}_{0}\times {\mathrm{Ins}}_{0}\right)\left({\mathrm{Glu}}_{\mathrm{mean}}\times {\mathrm{Ins}}_{\mathrm{mean}}\right)}$, where Glu_0_ represented the blood glucose level in fasting state and Glu_mean_/Ins_mean_ represented the average blood glucose and insulin values from the seven timepoints collected. The TyG index—a measure of non-insulin-based insulin resistance—was calculated as *ln* (triglycerides level at fasting (mg/dL) × blood glucose level at fasting (mg/dL)/2) according to the published literature [42].

### 2.6. Statistical Analysis

The differences in baseline characteristics and the five studied fat phenotypes (i.e., IPFD, IHFD, SMFD, VFV, SFV) between the three groups (NODAP, T2DM, and healthy controls) were evaluated using a one-way ANOVA and chi-square test. Data are presented as the median and interquartile range or frequency. The subsequent statistical analyses were conducted in two steps. First, to examine the associations between the five studied fat phenotypes and indices of insulin sensitivity (i.e., HOMA-IS, Raynaud index, Matsuda index, TyG index), a univariate and multiple variable linear regression analysis were conducted. Each index of insulin sensitivity was treated as a dependent variable. The following three models were constructed: (1) unadjusted, (2) adjusted for age and sex, and (3) adjusted for age, sex, and HbA1c. All data were reported as β coefficients with corresponding standard errors and *p* values. The most robust R^2^ metric was yielded for each association.

Second, to investigate the relative contributions of the five studied abdominal fat phenotypes to variance in each index of insulin sensitivity, relative importance analysis was conducted within each group using the “relaimpo” package [43]. This analysis involved constructing a multivariable linear regression model, yielding R^2^ metrics of all included independent variables explaining each index of insulin sensitivity, and depicting all the R^2^ metrics. All statistical analyses were conducted using SAS version 9.4 for Windows (SAS Institute, Cary, NC, USA) and R Studio version 3.6.1 (RStudio Inc., Boston, MA, USA). A two-sided *p* < 0.05 was deemed to be statistically significant.

## 3. Results

### 3.1. Characteristics of Study Participants

A total of 78 individuals were included, of whom 26 had NODAP, 20 had T2DM, and 32 were healthy controls. The median time since the last attack of pancreatitis was 18 months (interquartile range, 11.0–23.0 months) and 22 months (interquartile range, 13.5–42.5 months) in the NODAP group and the T2DM group, respectively. Other characteristics are presented in Table 1.

### 3.2. Abdominal Fat Phenotypes in the Study Groups

The mean ± standard deviation of IPFD (%) was 8.7 ± 2.1%, 9.6 ± 1.5%, and 7.5 ± 2.0% in the NODAP, T2DM, and healthy controls groups, respectively. There was a statistically significant difference in IPFD between the three groups (*p* = 0.001). 

The mean ± standard deviation of IHFD (%) was 7.4 ± 5.6%, 14.4 ± 20.0%, and 7.4 ± 6.0% in the NODAP, T2DM, and healthy controls groups, respectively. There was no statistically significant difference in IHFD between the three groups.

The mean ± standard deviation of SMFD (%) was 15.1 ± 7.4%, 17.0 ± 6.5%, and 14.1 ± 7.1% in the NODAP, T2DM, and healthy controls groups, respectively. There was no statistically significant difference in SMFD between the three groups.

The mean ± standard deviation of VFV (cm^3^) was 1494.5 ± 675.4 cm^3^, 1944.6 ± 820.3 cm^3^, and 1023.4 ± 691.4 cm^3^ in the NODAP, T2DM, and healthy controls groups, respectively. There was a statistically significant difference in VFV between the three groups (*p* < 0.001).

The mean ± standard deviation of SFV (cm^3^) was 2162.5 ± 687.9 cm^3^, 2461.4 ± 904.1 cm^3^, and 2247.4 ± 1066.7 cm^3^ in the NODAP, T2DM, and healthy controls groups, respectively. There was no statistically significant difference in SFV between the three groups.

### 3.3. Associations between Abdominal Fat Phenotypes and Indices of Insulin Sensitivity in the Study Groups

#### 3.3.1. Intra-Pancreatic Fat Deposition

In the NODAP group, IPFD was significantly associated with HOMA-IS in both the unadjusted (β = −0.186, *p* = 0.005) and two adjusted models (*p* = 0.011 in model 2; *p* = 0.024 in model 3). IPFD was significantly associated with the Raynaud index in both the unadjusted (β = −5.271, *p* = 0.033) and two adjusted models (*p* = 0.026 in model 2; *p* = 0.032 in model 3). IPFD was significantly associated with the Matsuda index in both the unadjusted (β = −7.451, *p* = 0.002) and two adjusted models (*p* = 0.011 in model 2; *p* = 0.022 in model 3). IPFD was significantly associated with the TyG index in both the unadjusted (β = 0.324, *p* = 0.002) and two adjusted models (*p* = 0.003 in model 2; *p* = 0.004 in model 3).

There was no statistically significant association (both in the unadjusted and adjusted models) between IPFD and the indices of insulin sensitivity in either the T2DM group or the healthy controls group (Table 2).

#### 3.3.2. Intra-Hepatic Fat Deposition 

In the NODAP group, IHFD was significantly associated with the TyG index in both the unadjusted (β = 0.014, *p* = <0.001) and two adjusted models (*p* = 0.001 in model 2; *p* = 0.001 in model 3).

There was no statistically significant association (both in the unadjusted and adjusted models) between IHFD and the indices of insulin sensitivity in either the T2DM group or the healthy controls group (Table 3).

#### 3.3.3. Skeletal Muscle Fat Deposition

In the T2DM group, SMFD was significantly associated with the Matsuda index in the age- and sex-adjusted model only (*p* = 0.016).

There was no statistically significant association (both in the unadjusted and adjusted models) between SMFD and the indices of insulin sensitivity in either the NODAP group or the healthy controls group (Table 4).

#### 3.3.4. Visceral Fat Volume 

In the NODAP group, VFV was significantly associated with HOMA-IS in the unadjusted model (β = −0.456, *p* = 0.031) only. VFV was significantly associated with the Matsuda index in the unadjusted model (β = −23.061, *p* = 0.012) and the age- and sex-adjusted model (*p* = 0.040). VFV was significantly associated with the TyG index in both the unadjusted (β = 1.200, *p* = < 0.001) and two adjusted models (*p* = 0.001 in model 2; *p* = 0.001 in model 3).

In the T2DM group, VFV was significantly associated with HOMA-IS in the unadjusted model (β = −0.150, *p* = 0.039) and the age- and sex-adjusted model (*p* = 0.037). VFV was significantly associated with the TyG index in both the unadjusted (β = 0.348, *p* = 0.024) and the age- and sex-adjusted model only (*p* = 0.031). 

There was no statistically significant association (both in the unadjusted and adjusted models) between VFV and the indices of insulin sensitivity in the healthy controls group (Table 5).

#### 3.3.5. Subcutaneous Fat Volume

In the NODAP group, SFV was significantly associated with HOMA-IS in both the unadjusted (β = −0.429, *p* = 0.041) and two adjusted models (*p* = 0.020 in model 2; *p* = 0.029 in model 3). SFV was significantly associated with the Matsuda index in both the unadjusted (β = −20.765, *p* = 0.039) and two adjusted models (*p* = 0.024 in model 2; *p* = 0.039 in model 3). SFV was significantly associated with the TyG index in two adjusted models (*p* = 0.012 in model 2; *p* = 0.016 in model 3).

There was no statistically significant association (both in the unadjusted and adjusted models) between SFV and the indices of insulin sensitivity in either the T2DM or the healthy controls group (Table 6).

### 3.4. Contribution of Abdominal Fat Phenotypes to Indices of Insulin Sensitivity in the Study Groups

**Figure 1:**
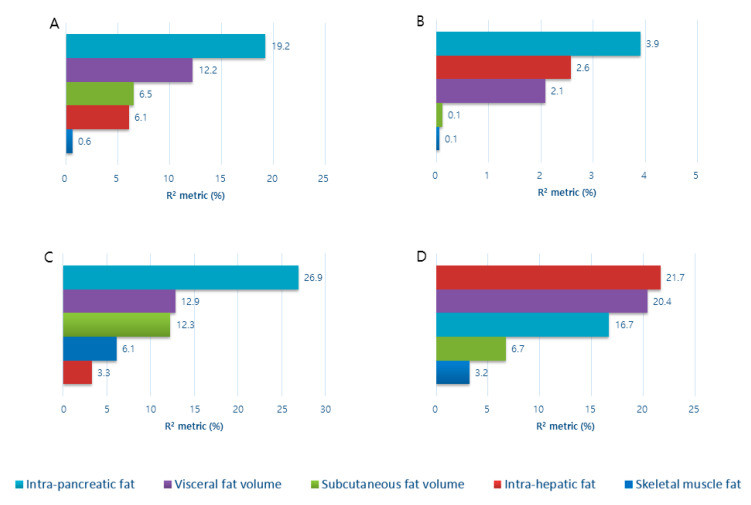
Contribution of the studied fat phenotypes to variance in indices of insulin sensitivity in non-obese individuals with NODAP. Data are presented as a percentage of the corresponding abdominal fat phenotype that explains the variance of (**A**) HOMA-IS; (**B**) Raynaud index; (**C**) Matsuda index; (**D**) TyG index. HOMA-IS: homeostasis model assessment estimate of insulin sensitivity; NODAP: new-onset prediabetes or diabetes after acute pancreatitis; TyG: triglyceride and glucose index.

In the NODAP group, the five abdominal fat phenotypes altogether explained 44.7% of the variance in HOMA-IS, 8.76%—Raynaud index, 61.4%—Matsuda index, and 68.6%—TyG index. Of the five abdominal fat phenotypes, IPFD significantly explained the most of variance in HOMA-IS (R^2^ = 19.2%, *p* = 0.03) and the Matsuda index (R^2^ = 26.9%, *p* = 0.05) (Figure 1). The other four abdominal fat phenotypes did not significantly contribute to the variance in the indices of insulin sensitivity (data not shown).

In the T2DM group, the five abdominal fat phenotypes altogether explained 26.5% of the variance in HOMA-IS, 19.6%—Raynaud index, 99.9%—Matsuda index, and 39.7%—TyG index. Of the five abdominal fat phenotypes, VFV significantly explained most of the variance in the Matsuda index (R^2^ = 48.3%, *p* = 0.02), followed by SFV (R^2^ = 19.6%, *p* = 0.04), SMFD (R^2^ = 19.1%, *p* = 0.04), IHFD (R^2^ = 9.9%, *p* = 0.05). IPFD did not significantly contribute to the variance in the Matsuda index (R^2^ = 3.1%, *p* = 0.33). None of the five abdominal fat phenotypes significantly contribute to the variance in the other indices of insulin sensitivity (data not shown).

In the healthy controls group, the five abdominal fat phenotypes altogether explained 11.1% of the variance in HOMA-IS, 10.2% in the Raynaud index, 12.1% in the Matsuda index, and 14.0% of the variance in the TyG index. None of the five abdominal fat phenotypes significantly contribute to the variance in any index of insulin sensitivity (data not shown).

## 4. Discussion

Insulin resistance, a long-established pathophysiological state linked to a spectrum of metabolic disorders, has mainly been investigated in populations with general obesity, and studies focusing on abdominal obesity have emerged only recently. This is the first study in the post-pancreatitis setting to investigate the associations between several abdominal fat phenotypes (derived from the state-of-the-art MR imaging and spectrospcopy) and several indices of insulin sensitivity (both insulin-based and non-insulin-based) in non-obese individuals. The key finding is that IPFD was significantly associated with all the studied indices of insulin sensitivity (in both fasting and postprandial states) in individuals with NODAP, consistently in both the unadjusted model and adjusted models. By contrast, IPFD was not significantly associated with any of the studied indices of insulin sensitivity in both individuals with T2DM and healthy controls.

Increased insulin resistance has been identified as a characteristic of NODAP [6,28,44]. Several clinical and experimental studies have shown a significant association between IPFD and insulin sensitivity in non-selected individuals with NODAP (i.e., both obese and non-obese based on BMI) [17,45]. The present study takes the field further by demonstrating a significant association between IPFD and insulin sensitivity specifically in non-obese individuals. The underlying mechanisms behind the observed association in NODAP are not fully understood, but one possible mechanism is increased IPFD due to elevated triglycerides [46,47]. This is supported by an animal study suggesting that a high-fat diet induces both interlobular and intralobular fat deposition [48]. Although there is little evidence on the role of fat in insulin sensitivity in the post-pancreatitis setting, a previous study showed a strong positive association between the levels of serum triglycerides and IPFD in non-selected individuals after AP (which, in theory, might have been attributed to a high BMI) [49]. However, given that the present study focused on non-obese individuals only, a pancreas-centered mechanism is likely to explain increased insulin resistance in individuals with NODAP. The possible pancreas-centered mechanism is fatty replacement of the pancreas triggered by recurrent insults to the organ [50]. Recurrent or low-grade persistent inflammation of the pancreas may facilitate fatty replacement in the damaged pancreatic acinar cells via acinar-to-adipocyte transdifferentiation, subsequently leading to IPFD [51]. Further, recurrent attacks of AP are known to be associated with a significantly increased risk of NODAP [52]. Taken together, dysfunction in the insulo-acinar axis (due to recurrent acinar cell damage and subsequent fatty replacement) [53] may result in β-cell dysfunction, death, or dedifferentiation [54,55], leading to increased insulin resistance and development of NODAP. 

The present study adds to the existing literature on factors that characterize NODAP. Several studies have demonstrated that there are differences between NODAP and T2DM in the gut hormone response [8], blood glucose control [9], risk of pancreatic cancer [10], risk of mortality [11], and survival benefit following the use of common antidiabetic medications [12,13]. To date, only one study has linked IPFD to insulin sensitivity in individuals with NODAP [26]. The authors have demonstrated that a fasting state index of insulin sensitivity (i.e., Raynaud index) was significantly inversely associated with IPFD in individuals with NODAP [26]. In contrast, none of the indices of insulin sensitivity were associated with IPFD in healthy individuals [26]. Although that study suggested that the relationship between IPFD and insulin sensitivity might play a role in the pathogenesis of NODAP, it is possible that general obesity confounded the observed association in individuals with NODAP. Moreover, individuals with NODAP were compared with healthy individuals only, and individuals with T2DM were not included in that study. In the present study, IPFD was associated with both fasting state indices of insulin sensitivity (i.e., HOMA-IS, Raynaud index, TyG index) and postprandial state index of insulin sensitivity (i.e., Matsuda index) in non-obese individuals with NODAP. Further, both insulin-based (i.e., HOMA-IS, Raynaud, Matsuda) and non-insulin-based (i.e., TyG) indices were significantly associated with IPFD in non-obese individuals with NODAP, whereas none of the associations were significant in non-obese individuals with T2DM. Recent studies have advocated the use of TyG index as a reliable and simple biomarker of insulin resistance in clinical practice since this index does not require quantification of insulin and may be reliably used in patients treated with insulin [42,56]. This index has also been shown to be a predictor of diabetes as it is significantly associated with the risk of incident diabetes [57]. Taken together, the above findings strongly suggest that increased insulin resistance in NODAP may be driven by IPFD and not general or visceral obesity (as it is the case in T2DM). 

There are a number of limitations in the present study that need to be acknowledged. First, because this study was cross-sectional, a causal role of IPFD in insulin resistance and NODAP cannot be established. However, to date, no longitudinal study on changes in IPFD has been conducted in individuals with NODAP. A prospective longitudinal study is now warranted to address this issue unequivocally. Second, the “gold standard” for the determination of insulin traits (i.e., the hyperinsulinemic–euglycemic clamp) [58] was not used in this study. Future studies may benefit from employing it in exploring the associations between IPFD and insulin traits in the post-pancreatitis setting. Third, insulin secretion was not investigated in the present study. However, in an earlier clinical study that investigated the associations between IPFD and nine indices of insulin secretion in individuals NODAP using a step-wise multivariable analysis, none of the indices of insulin secretion appeared in the final model [26], indicating that insulin sensitivity is a more relevant trait than insulin secretion when it comes to their associations with IPFD in individuals with NODAP. Fourth, although we excluded participants with chronic pancreatitis, recurrent attacks of AP might have affected the association between the indices of insulin sensitivity and IPFD [32]. Given that the number of participants with recurrent bouts was small, a meaningful statistical analysis was not possible. Moreover, there is no evidence that recurrent bouts would affect the other studied fat phenotypes. Last, no genetic data were available. It has been shown that genes are associated with general obesity and, more recently, ectopic fat depots [59]. Genetic factors may need to be considered in future studies on the association between IPFD and insulin sensitivity.

In conclusion, IPFD was positively associated with insulin resistance in non-obese individuals with NODAP (but not in non-obese individuals with T2DM or healthy controls). Moreover, of the studied abdominal fat phenotypes, IPFD explained the most variance in both fasting (HOMA-IS) and postprandial (Matsuda index) indices of insulin sensitivity. Longitudinal studies are now warranted to investigate the cause and effect relationship between IPFD and insulin resistance in individuals with NODAP.

## Figures and Tables

**Table 1:** Characteristics of the study groups.

Characteristic	Healthy Controls (*n* = 32)	T2DM (*n* = 20)	NODAP (*n* = 26)	*p* *
Age (years)	46.0 (29.5–63.0)	59.5 (49.5–72.0)	58.0 (47.0–66.0)	**0.036**
Men, n (%)	19 (54.3)	16 (76.2)	19 (70.1)	0.195
Body mass index (kg/m^2^)	23.6 (21.6–26.6)	26.4 (24.2–28.1)	24.7 (22.3–27.1)	**0.009**
Triglycerides (mmol/L)	0.9 (0.6–1.2)	1.5 (0.9–2.0)	1.5 (1.1–2.4)	**0.044**
Total cholesterol (mmol/L)	4.5 (3.7–5.5)	3.6 (4.5–5.4)	5.0 (4.1–5.4)	0.431
HDL cholesterol (mmol/L)	1.3 (0.9–1.8)	1.3 (1.0–1.6)	1.3 (1.1–1.6)	0.791
LDL cholesterol (mmol/L)	2.7 (2.0–3.3)	2.8 (2.1–3.2)	2.6 (2.3–3.4)	0.624
Glycated hemoglobin A1c (mmol/mol)	33.0 (31.0–35.0)	41.0 (38.5–9.5)	38.5 (36.0–41.1)	**<0.001**
Fasting plasma glucose (mmol/L)	4.8 (4.2–5.3)	10.8 (8.9–19.4)	8.4 (4.8–14.3)	**<0.001**

NODAP: new-onset prediabetes or diabetes after acute pancreatitis; T2DM: type 2 prediabetes or diabetes mellitus; data are presented as median and interquartile range or percentage; * *p* values are from one-way ANOVA; statistically significant values (*p* less than 0.05) are in bold.

**Table 2:** Associations between intra-pancreatic fat deposition and indices of insulin sensitivity in non-obese individuals.

Index	Healthy Controls				T2DM				NODAP			
	β	S.E.	*p*	R^2^	β	S.E.	*p*	R^2^	β	S.E.	*p*	R^2^
HOMA-IS												
Model 1	−0.057	0.109	0.608	0.013	−0.035	0.041	0.406	0.037	−0.186	0.060	**0.005**	0.285
Model 2	−0.092	0.116	0.438		−0.038	0.043	0.398		−0.204	0.074	**0.011**	
Model 3	−0.080	0.136	0.565		−0.015	0.042	0.721		−0.199	0.082	**0.024**	
Raynaud index												
Model 1	0.030	0.244	0.902	0.001	−0.384	0.714	0.597	0.015	−5.271	2.330	**0.033**	0.176
Model 2	0.074	0.249	0.768		−0.453	0.756	0.557		−6.726	2.806	**0.026**	
Model 3	0.102	0.272	0.711		−0.274	0.798	0.736		−7.147	3.101	**0.032**	
Matsuda index												
Model 1	1.320	5.291	0.806	0.004	0.714	2.529	0.787	0.023	−7.451	2.016	**0.002**	0.477
Model 2	2.147	5.814	0.718		0.459	3.149	0.891		−8.648	2.561	**0.011**	
Model 3	1.503	6.447	0.820		2.096	1.681	0.303		−7.904	3.003	**0.022**	
TyG												
Model 1	0.036	0.056	0.532	0.017	0.178	0.159	0.277	0.065	0.324	0.091	**0.002**	0.357
Model 2	0.048	0.049	0.338		0.182	0.170	0.299		0.354	0.106	**0.003**	
Model 3	0.032	0.053	0.547		0.058	0.158	0.720		0.379	0.11	**0.004**	

HOMA-IS: homeostasis model assessment estimate of insulin sensitivity; NODAP: new-onset prediabetes or diabetes after acute pancreatitis; T2DM: type 2 prediabetes or diabetes mellitus; TyG: triglyceride and glucose index; data are presented as β coefficients, standard errors, *p* values (from linear regression), and R2 values (from crude analysis); statistically significant values (*p* less than 0.05) are in bold; Model 1: unadjusted model; Model 2: adjusted for age and sex; Model 3: adjusted for age, sex, and glycated hemoglobin A1c.

**Table 3:** Associations between intra-hepatic fat deposition and indices of insulin sensitivity in non-obese individuals.

Index	Healthy Controls				T2DM				NODAP			
	β	S.E.	*p*	R^2^	β	S.E.	*p*	R^2^	β	S.E.	*p*	R^2^
HOMA-IS												
Model 1	0.011	0.034	0.740	0.005	−0.002	0.003	0.589	0.017	−0.051	0.024	0.046	0.150
Model 2	0.005	0.036	0.888		−0.002	0.004	0.625		−0.048	0.027	0.087	
Model 3	−0.009	0.042	0.833		−0.001	0.003	0.681		−0.048	0.027	0.085	
Raynaud index												
Model 1	0.072	0.077	0.358	0.033	−0.035	0.058	0.555	0.020	−1.216	0.920	0.198	0.065
Model 2	0.086	0.077	0.277		−0.032	0.060	0.596		−1.336	1.018	0.202	
Model 3	0.095	0.086	0.279		−0.029	0.061	0.637		−1.341	1.035	0.208	
Matsuda index												
Model 1	−0.687	1.380	0.625	0.015	0.312	0.296	0.341	0.181	−1.768	0.904	0.069	0.203
Model 2	−0.564	1.472	0.707		0.491	0.555	0.441		−1.482	1.092	0.198	
Model 3	−0.411	1.782	0.821		0.053	0.467	0.920		−1.586	1.084	0.169	
TyG												
Model 1	−0.013	0.020	0.527	0.016	0.007	0.013	0.603	0.016	0.014	0.031	**<0.001**	0.461
Model 2	−0.008	0.018	0.674		0.007	0.014	0.637		0.128	0.034	**0.001**	
Model 3	0.001	0.020	0.945		0.005	0.012	0.659		0.129	0.034	**0.001**	

HOMA-IS: homeostasis model assessment estimate of insulin sensitivity; NODAP: new-onset prediabetes or diabetes after acute pancreatitis; T2DM: type 2 prediabetes or diabetes mellitus; TyG: triglyceride and glucose index; data are presented as β coefficients, standard errors, *p* values (from linear regression), and R^2^ values (from crude analysis); statistically significant values (*p* less than 0.05) are in bold; Model 1: unadjusted model; Model 2: adjusted for age and sex; Model 3: adjusted for age, sex, and glycated hemoglobin A1c.

**Table 4:** Associations between skeletal muscle fat deposition and indices of insulin sensitivity in non-obese individuals.

Index	Healthy Controls				T2DM				NODAP			
	β	S.E.	*p*	R^2^	β	S.E.	*p*	R^2^	β	S.E.	*p*	R^2^
HOMA-IS												
Model 1	−0.004	0.031	0.894	0.001	0.002	0.010	0.874	0.001	0.004	0.020	0.846	0.002
Model 2	−0.045	0.043	0.309		0.014	0.018	0.464		0.008	0.035	0.816	
Model 3	−0.040	0.046	0.399		0.006	0.018	0.722		0.019	0.036	0.604	
Raynaud index												
Model 1	−0.064	0.068	0.358	0.033	0.014	0.175	0.938	<0.001	0.170	0.723	0.816	0.002
Model 2	−0.031	0.096	0.754		0.278	0.313	0.387		0.822	1.262	0.521	
Model 3	−0.028	0.100	0.782		0.223	0.325	0.503		1.066	1.324	0.430	
Matsuda index												
Model 1	−1.420	1.266	0.279	0.072	1.062	0.957	0.310	0.170	0.592	0.819	0.557	0.024
Model 2	−1.919	2.157	0.389		4.140	1.022	**0.016**		0.628	1.520	0.686	
Model 3	−2.067	2.504	0.243		2.943	1.830	0.206		1.118	1.169	0.490	
TyG												
Model 1	−0.012	0.018	0.556	0.014	−0.0363	0.041	0.397	0.042	−0.031	0.031	0.322	0.041
Model 2	0.020	0.022	0.375		−0.123	0.079	0.139		−0.014	0.051	0.793	
Model 3	0.015	0.023	0.504		−0.092	0.069	0.203		−0.024	0.054	0.665	

HOMA-IS: homeostasis model assessment estimate of insulin sensitivity; NODAP: new-onset prediabetes or diabetes after acute pancreatitis; T2DM: type 2 prediabetes or diabetes mellitus; TyG: triglyceride and glucose index; data are presented as β coefficients, standard errors, *p* values (from linear regression), and R^2^ values (from crude analysis); statistically significant values (*p* less than 0.05) are in bold; Model 1: unadjusted model; Model 2: adjusted for age and sex; Model 3: adjusted for age, sex, and glycated hemoglobin A1c.

**Table 5:** Associations between visceral fat volume and indices of insulin sensitivity in non-obese individuals.

Index	Healthy Controls				T2DM				NODAP			
	β	S.E.	*p*	R^2^	β	S.E.	*p*	R^2^	β	S.E.	*p*	R^2^
HOMA-IS												
Model 1	−0.018	0.292	0.950	<0.001	−0.150	0.068	**0.039**	0.206	−0.456	0.120	**0.031**	0.172
Model 2	0.022	0.333	0.949		−0.186	0.081	**0.037**		−0.444	0.229	0.067	
Model 3	0.144	0.375	0.705		−0.154	0.080	0.072		−0.409	0.237	0.099	
Raynaud index												
Model 1	0.238	0.069	0.732	0.005	−2.224	1.211	0.082	0.151	−10.998	7.605	0.161	0.077
Model 2	0.598	0.742	0.428		−2.751	1.464	0.078		−12.773	8.673	0.154	
Model 3	0.797	0.829	0.347		−2.542	1.537	0.118		−12.364	9.067	0.187	
Matsuda index												
Model 1	5.535	12.654	0.668	0.012	−7.577	3.994	0.107	0.375	−23.061	8.034	**0.012**	0.355
Model 2	4.132	14.863	0.785		−9.099	5.814	0.193		−28.311	12.408	**0.040**	
Model 3	2.477	16.795	0.885		−2.757	5.570	0.655		−27.023	13.948	0.077	
TyG												
Model 1	0.171	0.176	0.341	0.035	0.348	0.107	**0.024**	0.254	1.200	0.249	**<0.001**	0.491
Model 2	0.062	0.175	0.725		0.744	0.316	**0.031**		1.130	0.287	**0.001**	
Model 3	−0.035	0.191	0.858		0.566	0.288	0.068		1.129	0.300	**0.001**	

HOMA-IS: homeostasis model assessment estimate of insulin sensitivity; NODAP: new-onset prediabetes or diabetes after acute pancreatitis; T2DM: type 2 prediabetes or diabetes mellitus; TyG: triglyceride and glucose index; data are presented as β coefficients, standard errors, *p* values (from linear regression), and R^2^ values (from crude analysis); statistically significant values (*p* less than 0.05) are in bold; Model 1: unadjusted model; Model 2: adjusted for age and sex; Model 3: adjusted for age, sex, and glycated hemoglobin A1c.

**Table 6:** Associations between subcutaneous fat volume and indices of insulin sensitivity in non-obese individuals.

Index	Healthy Controls				T2DM					NODAP			
	β	S.E.	*p*	R^2^	β	S.E.	*p*	R^2^	β		S.E.	*p*	R^2^
HOMA-IS													
Model 1	0.198	0.196	0.323	0.044	0.068	0.071	0.354	0.045	−0.429		0.199	**0.041**	0.041
Model 2	0.167	0.212	0.441		0.070	0.090	0.448		−0.506		0.202	**0.020**	
Model 3	0.156	0.217	0.480		0.037	0.085	0.669		−0.4882		0.206	**0.029**	
Raynaud index													
Model 1	0.021	0.465	0.964	<0.001	1.191	1.228	0.344	0.047	−14.182		7.277	0.062	0.132
Model 2	−0.082	0.485	0.867		1.253	1.153	0.428		−15.249		7.745	0.061	
Model 3	−0.089	0.497	0.859		1.001	1.593	0.539		−14.956		8.003	0.075	
Matsuda index													
Model 1	3.809	9.310	0.688	0.010	5.884	4.586	0.247	0.215	−20.765		9.198	**0.039**	0.254
Model 2	4.974	10.007	0.627		6.144	6.212	0.379		−22.220		8.726	**0.024**	
Model 3	5.841	10.500	0.588		2.925	4.375	0.543		−21.074		9.105	**0.039**	
TyG													
Model 1	0.001	0.121	0.994	<0.001	−0.341	0.272	0.225	0.081	0.629		0.325	0.065	0.135
Model 2	0.075	0.112	0.509		−0.352	0.344	0.322		0.812		0.295	**0.012**	
Model 3	0.086	0.111	0.444		−0.193	0.305	0.536		0.797		0.304	**0.016**	

HOMA-IS: homeostasis model assessment estimate of insulin sensitivity; NODAP: new-onset prediabetes or diabetes after acute pancreatitis; T2DM: type 2 prediabetes or diabetes mellitus; TyG: triglyceride and glucose index; data are presented as β coefficients, standard errors, *p* values (from linear regression), and R^2^ values (from crude analysis); statistically significant values (*p* less than 0.05) are in bold; Model 1: unadjusted model; Model 2: adjusted for age and sex; Model 3: adjusted for age, sex, and glycated hemoglobin A1c.
